# Piloting an ecological momentary assessment block design to examine suicide outcomes and mechanisms in a caring contacts trial

**DOI:** 10.1016/j.invent.2026.100995

**Published:** 2026-09-30

**Authors:** Keyne C. Law, Andrew H. Rogers, Annie Hoang, Anna Evanson, Barbara Wright, Katherine Anne Comtois

**Affiliations:** a Department of Clinical Psychology, College of Applied and Natural Sciences, Seattle Pacific University, 3307 Fifth Avenue West, Seattle, WA, 98119, United States; b Division of Behavioral Medicine, Department of Medicine, Jacobs School of Medicine and Biomedical Sciences, University at Buffalo, State University of New York, 462 Grider Street, Buffalo, New York, 14215, United States; c Department of Psychiatry & Behavioral Sciences, School of Medicine, University of Washington, 1959 NE Pacific Street, Seattle, WA, 98195, United States; d Department of Pediatric Oncology, Roswell Park Comprehensive Cancer Center, Elm & Carlton Streets, Buffalo, NY, 14263, United States

**Keywords:** Ecological momentary assessment, Measurement burst, Suicide, Caring contacts, Intervention, Mechanisms

## Abstract

Ecological momentary assessment (EMA) can capture short-term fluctuations in suicidal thoughts and related processes that are obscured by traditional pre-post assessments, but sustained intensive sampling may impose substantial participant burden. Measurement-burst designs address this tradeoff by repeating brief periods of intensive assessment across longer timescales. We piloted a repeated EMA block design consisting of one week of EMA per month across three months within a Caring Contacts intervention study. The current pilot study included 30 participants receiving outpatient mental health services; all received Caring Contacts and completed EMA for one/month over 3 months with items focused on variability in suicide outcomes (desire to live, desire to die, suicide urges) and mechanisms through which caring contacts could impact these outcomes (perceived burdensomeness, entrapment, mattering, social & personal responsibility), and a qualitative interview. Results provide support for the feasibility and acceptability of utilizing EMA in the context of Caring Contacts delivery and provide initial support for the use of EMA to capture dynamic mechanisms of suicide outcomes. Repeated EMA blocks offer a promising approach for examining suicide-related processes across timescales longer than those typically captured by a single period of intensive sampling while limiting the burden of continuous EMA. Larger studies are needed to determine whether repeated EMA blocks provide meaningful advantages over less intensive assessment schedules for detecting change in suicide-related mechanisms and outcomes.

## Highlights

• Repeated EMA blocks can capture suicide-related processes across longer timescales
• A one-week-per-month EMA block design showed promising feasibility and acceptability
• EMA blocks captured within-person variability in suicide outcomes and proposed mechanisms

## 1 Introduction

Psychological interventions are increasingly delivered outside the clinic. Remotely delivered and technology-mediated approaches (i.e., internet-based treatment programs, mobile applications, and brief contact interventions such as caring messages) now account for a substantial share of the intervention landscape (Vernmark et al., 2024). Research designs that can capture proximal and distal, acute and gradual changes are needed to understand and evaluate mechanisms of change of such interventions. Standard trial assessment collects a small number of retrospective summary reports at widely spaced protocol-defined occasions. Unfortunately, this approach may be sampling too slowly to capture a process that changes within days and do so under conditions that bear little resemblance to those in which the intervention operates.

To understand how interventions produce change, we need methods that capture the complex, dynamic patterns of suicidal ideation that can fluctuate significantly from day to day or even within a single day (Bryan et al., 2019, Bryan et al., 2020; Bryan and Rudd, 2018; Coppersmith et al., 2023; Hallensleben et al., 2018). Traditional pre-post assessments, although easy to administer, can approximate but are limited in their ability to directly observe change in the causal mechanisms through which the intervention exerts its effects. Intensive time sampling methods, like Ecological Momentary Assessment (EMA) (Bryan, 2019), have helped researchers capture moment-to-moment variations in suicidal ideation (Ballard et al., 2021) and examine how suicide-related outcomes evolve in response to changing contexts, stressors, and recovery phases (Kivelä et al., 2022; Kleiman and Nock, 2018; Rabasco and Sheehan, 2022; Sedano-Capdevila et al., 2021). However, these studies often only capture a small window of time (5 to 42 days) and may not fully reflect the variability of suicide risk across longer intervention periods. On the other hand, continuous EMA monitoring for longer periods of time risks overwhelming participants, leading to attrition.

Measurement burst designs (Sliwinski, 2008; Stawski et al., 2015), often used in developmental research, extend EMA by administering short bursts of EMA surveys in repeated intervals over long spans of time (i.e. years). This approach captures micro-level dynamics within each burst and allows investigators to observe how dynamic processes change across broader time scales. Repeated bursts allow investigators to separate stable between-person differences in a within-person process from genuine change in that process over time, and both from measurement error (Sliwinski, 2008). Thus, EMA bursts can characterize whether and how within-person processes vary across longer timescales that would not be observable within a single period of intensive sampling. For example, Sliwinski and colleagues (2009) found substantial within-person variability in stress reactivity across repeated measurement bursts, with 73% of the reliable variance in reactivity occurring within individuals across bursts. Moreover, this burst-to-burst variability was systematically associated with concurrent fluctuations in perceived stress, suggesting that differences across bursts may reflect meaningful changes in individuals' psychosocial contexts rather than measurement error alone. Existing studies using EMA burst designs have varied considerably in both the duration and spacing of bursts. Intensive sampling periods have ranged from 3 to 4 days (Biller et al., 2025; Ponnada et al., 2022) to two weeks (Helle et al., 2023) and have been repeated at intervals ranging from every two weeks (Prochnow et al., 2025), every three weeks (Helle et al., 2023), and every four weeks (Biller et al., 2025) to substantially longer intervals, such as every 5 years (Carstensen et al., 2011). Other designs time bursts to theoretically or clinically meaningful periods rather than fixed intervals. For example, some clinical trials administer bursts corresponding to different segments of the intervention such as pre-, mid-, and post-treatment (Miller-Chagnon et al., 2024).

Adapting the logic of EMA burst designs, we piloted a repeated EMA block design in which participants completed a one-week block of EMA during the first week of each month (approximately 28–31 days apart vs. fixed time intervals, e.g., every 4 weeks) across a three-month period. Because both the scheduling and the content of each assessment period were fixed in advance, such that survey items were delivered on a calendar-anchored schedule known to participants from enrollment, we refer to these periods as blocks rather than bursts. Each block is the unit within which within-person dynamics can be estimated and across which change over the course of the trial is evaluated. Blocks were anchored to the start of each calendar month rather than to a fixed interval on the reasoning that a recurring, predictable landmark would be easier for participants to anticipate and accommodate across a multi-month protocol. The design is a fixed-schedule variant of a measurement burst design rather than a distinct one; the terminology should be considered descriptive as it carries no methodological claim. The goal of this design was to enable the collection of frequent, high-quality data and the evaluation of mechanistic processes across treatment phases while balancing participant feasibility in a clinical trial of Caring Contacts.

This pilot study examined the feasibility and preliminary effectiveness of using this repeated EMA block design over a 3-month period to monitor suicide-related mechanisms and outcomes during the delivery of Caring Contacts to inform the design of a larger Caring Contacts clinical trial (Wright et al., 2025). First, we evaluated how data gathered through EMA blocks aligns with data collected using less intensive pre-post sampling approaches, assessing whether the additional labor, resource investment, and participant burden required are justified by their ability to capture meaningful month-to-month changes. Given the dynamic nature of suicidal ideation, we then tested whether we can effectively detect variability in suicide-related constructs using EMA blocks. If able to capture variability over months, EMA blocks can provide insights into how suicide risk evolves and how interventions like Caring Contacts influence these dynamics. Specifically, we investigated whether variability differs between individuals with and without suicidal ideation (SI) and whether Caring Contacts influenced changes in these mechanisms over time. We also examined perceived burdensomeness, entrapment, mattering, social responsibility, and personal responsibility, which are theorized to mediate the effects of Caring Contacts on suicide risk. Finally, we evaluated our ability to automate the EMA process to reduce human involvement while maintaining high participant engagement by assessing the consistency, usability, feasibility, and acceptability of EMA blocks through qualitative feedback from participants. Altogether, we hope that this study will help fill a methodological gap in suicide research by testing a scalable approach to capturing dynamic, temporal changes in suicide-related mechanisms.

### 1.1 The caring contacts intervention

Caring Contacts (CC), the delivery of brief, supportive messages via postal mail, email, or text at regular intervals, is one of the few low-cost interventions demonstrating protective effects against suicide (Luxton et al., 2012; Reger et al., 2017). Over four decades of research have found that the delivery of CC can reduce suicide deaths, attempts, ideation, and hospitalizations, with the most consistent result in reducing the risk of a suicide attempt (Comtois et al., 2019; Radin et al., 2023; Skopp et al., 2023; Steinberg et al., 2024). Prior CC trials have primarily recruited individuals receiving crisis or mental health care, including patients following psychiatric hospitalization or emergency care and individuals identified through military medical and mental health settings (Ward-Ciesielski and Wilks, 2020). The evidence base also includes studies conducted specifically with active-duty military personnel and Veterans (Ward-Ciesielski and Wilks, 2020). Despite these findings, however, a gap remains in understanding the psychological and behavioral processes through which CC reduces suicide risk. This limits our insights into how and when is best to implement it.

It is often hypothesized that CCs reduce suicide risk by addressing an individual's sense of connectedness. In the context of suicide, we conceptualize connectedness as a multidimensional construct encompassing interpersonal and intrapersonal experiences that may be responsive to expressions of care. Perceived burdensomeness, the perception that one is a burden on others or that others would be better off without them, along with thwarted belongingness, a sense of social disconnection and unmet need to belong, are posited by the Interpersonal Theory of Suicide (Joiner, 2007) to contribute to the development of suicidal desire. CC may mitigate these interpersonal experiences by providing unsolicited expressions of care and connection, potentially communicating to recipients that they are valued and remain connected to others even without initiating contact themselves. In the existing body of research on the interpersonal theory of suicide, perceived burdensomeness has consistently been found to have a stronger, more robust association with suicidal ideation than thwarted belongingness (Radin et al., 2023; Silvia et al., 2014). Given the need to limit participant burden in the EMA protocol, we therefore prioritized perceived burdensomeness as the interpersonal mechanism assessed in the present study and did not assess thwarted belongingness. Mattering, the belief that one is significant and valued by others (Elliott et al., 2004; Flett, 2022; Tonini et al., 2025), has also been linked to the reduced likelihood of considering suicide (Elliott et al., 2005) and may be strengthened by the unsolicited expressions of care and concern communicated through CC. By conveying that someone is thinking about and cares about the recipient without requiring the recipient to first seek support, these messages may reinforce a sense of being noticed and valued by others. Entrapment, the perception of being trapped in an aversive situation from which there is no perceived escape, is central to the Integrated Motivational-Volitional Model of suicide and related escape-based theories (O'Connor and Kirtley, 2018). CC may reduce feelings of entrapment by reminding recipients of connections and sources of support outside their immediate circumstances, potentially broadening perceived alternatives when circumstances feel inescapable. Lastly, a sense of responsibility and commitment to others has been identified as a potential reason for living and has been associated with lower suicidal ideation and suicidal cognitions (Linehan et al., 1983; McLaren, 2022; Rozek et al., 2021). In this study, we conceptualize this sense of responsibility in terms of personal and social responsibility. Personal responsibility refers to a sense of accountability for one's choices and behaviors, including their consequences for oneself and others, whereas social responsibility refers to a commitment to making choices and engaging in behaviors that promote the well-being of others and society (Arslan and Wong, 2021). CC may reinforce these interpersonal commitments by affirming interpersonal connection and reminding recipients of their relationships and capacity to remain engaged with others.

## 2 Methods

### 2.1 Participants and recruitment

Participants consisted of psychiatry patients from an outpatient psychiatry clinic (OPC) and veterans from a local veteran service organization. These sites represent key use cases for CC implementation. Prospective participants were identified to potentially benefit from receiving CC by site staff members and referred to the study by their site contact (either their case worker or social worker). To be eligible for the study, participants were required to be 18 or older at the time of enrollment, own a cell phone, willing to receive CC via text message, and willing to complete EMA surveys for a week each month of follow-up. To prevent fraudulent responses, the two sites were required to verify that they had invited the participant to the study before the participant was compensated for completing the baseline survey.

### 2.2 Procedures

Participants who were eligible for the study after completing the initial screening survey were provided with a secure link to access the Research Electronic Data Capture (REDCap) study flow used for data collection. After completing the informed consent process, participants were given the option to continue with the survey or be emailed a link for completion at a later date. Participants completed a baseline survey and then registered their contact information to receive 3 months of CCs and EMAs. They were informed at this time that they will be receiving a series of caring and supportive text messages every few weeks for three months. They were also told that responding was optional and that they would receive a response if they chose to reply.

During the first 7 days of each month, participants received invitation links via text message and email to complete EMA assessments at three randomly selected times for a total of 63 possible EMA assessments across the three-month study period. The specific EMA items, including their temporal framing and response options, are described below. Consistent with typical EMA protocols (Bell et al., 2024; Hartson et al., 2023), EMA prompts expired after 35 min to encourage responses proximal to the time of prompting and prevent multiple assessments from accumulating and being completed simultaneously. Each EMA generally took between 30 s and 3 min to complete. At the end of the 3 months, participants were automatically sent a follow-up survey via REDCap and given a 2-month window to complete the follow-up survey. Given that each site chose to implement different CC care plans, participants at the OPC site were invited to complete the follow-up survey 5 days before the month 3 EMA. In comparison, participants in the VSO site were invited to complete the follow-up survey 2 days after finishing the month 3 EMA. A random subset of the participants were asked to complete a qualitative interview on the usability, feasibility, and acceptability of the EMA blocks and study methods. Participants were invited to participate in the qualitative interviews in a randomized order, continuing until either all participants were contacted or data saturation was achieved. Participants completed the qualitative interviews between 4 and 8 months after completing the quantitative follow-up assessment.

All study procedures were reviewed and approved by the Institutional Review Board at the institution where the study was conducted and adhered to recognized ethical standards for research involving human participants (e.g., the Declaration of Helsinki). Participants were compensated with $25 for completing the baseline survey, $25 for completing the follow-up surveys, and between $2 and $10 for completing each EMA block. For each EMA block, they were paid $2 if they completed any EMAs, $4 if they completed more than 5 but less than 50% of EMAs, $6 if they completed 50–89% of EMAs, and $10 if they completed 90% or more of EMAs. Finally, participants were paid $50 if they participated in the qualitative interview.

### 2.3 Caring contacts

CC was delivered using a web-based application developed by the senior author (KC) to send, receive, and respond to messages with participants (Davidson et al., 2017). The application has the capability to preschedule messages, modify message content and delivery date/time, display conversation history, and document notes and alerts within each participant's record. Study team members were notified when a participant replied and if they needed to follow up on unread messages. Each site (OPC and VSO) was responsible for making decisions regarding their CC delivery following guidance from existing research on CC. Their preferences were then programmed into the web-based application, and their staff were responsible for initiating the delivery of the CC. Site staff were also responsible for providing responses during business hours, offering support and resources based on participant requests. Allowing variability in how each site implemented CC served as a natural experiment that provided insight into the feasibility and adaptability of CC delivery across each setting, which then directly informed final protocol design for the clinical trial (Wright et al., 2025).

#### 2.3.1 OPC

The OPC site sent up to 10 messages (8 standard, 1 birthday, and 1 fall holiday). Standard messages were sent approximately weekly, on study days 1, 8, 15, 22, 36, 50, 64, and 78 (See Fig. 1A). Messages were signed using a group name (e.g., Your Care Team), not a specific individual's name, and sent messages on Monday, Tuesday, and Wednesday. If a recipient sent a reply indicating distress or suicidality, OPC followed internal protocols with available clinical and medical staff when necessary.

**Fig. 1A:**
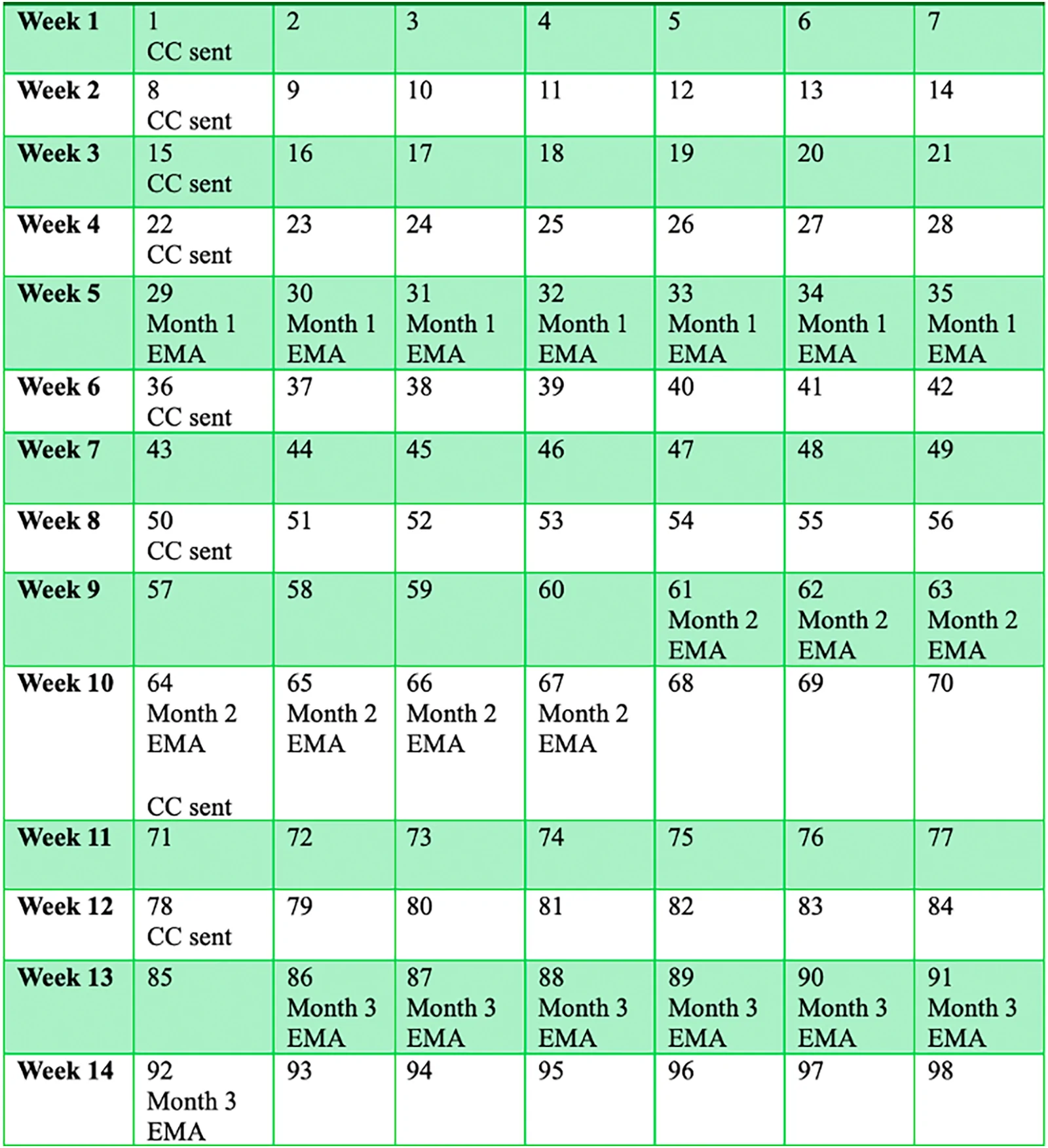
OPC Caring Contacts (CC) and EMA Schedule. **Note:** This Caring Contacts schedule does not include the additional two Caring Contacts for the participant's birthday and Thanksgiving.

#### 2.3.2 VSO

The VSO site sent up to 15 messages (12 standard, 1 birthday, 1 Veteran's Day, and 1 fall holiday). Standard messages were also sent approximately weekly, although the interval between messages varied over the course of the intervention (study days 1, 7, 15, 23, 33, 45, 53, 60, 67, 75, 82, and 91; See Fig. 1B). Like OPC, messages were signed using a group name (e.g., Your Care Team), not a specific individual's name. If a recipient sent a reply indicating distress or suicidality, VSO staff offered a warm handoff to 988 or directed the recipient to go to the emergency room.

**Fig. 1B:**
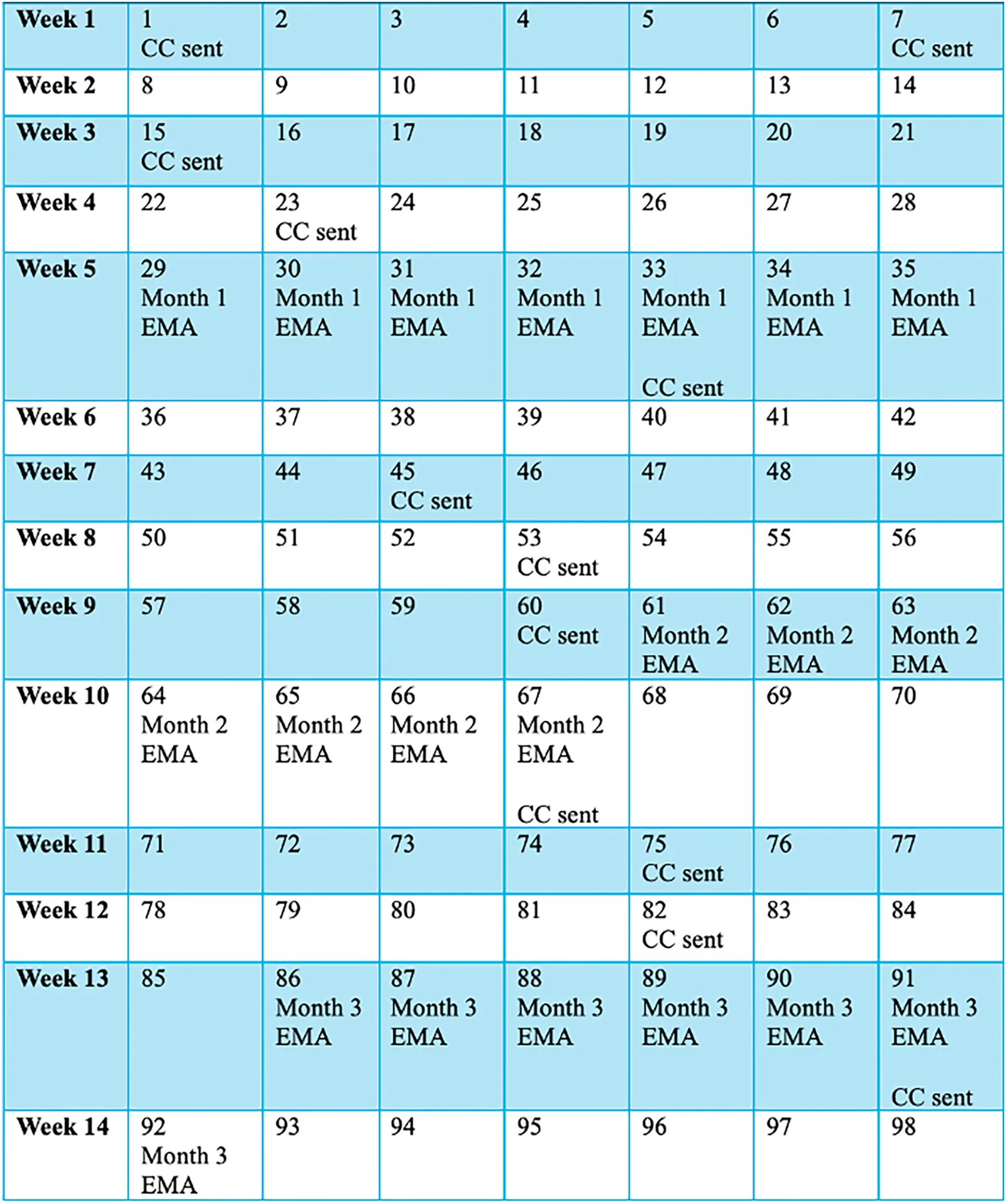
VSO Caring Contacts (CC) and EMA Schedule. **Note:** This Caring Contacts schedule does not include the additional three Caring Contacts for the participant's birthday, Veteran's Day, and Thanksgiving.

To facilitate assessment of CC mechanisms, the messages were scheduled to vary across the 3 EMA blocks. Timing and days were varied for delivering CC to create a more natural communication flow and prevent interactions from seeming automated or like spam (Ammerman et al., 2020; Reger et al., 2019, Reger et al., 2023). In this study, only three of the VSO site's regular messages and 1 of the clinic site's regular messages were scheduled to fall during an EMA block. This was done to avoid conditioning participants to expect CC messages during an EMA block. For messages that did align with the EMA blocks, the messages' impact could be measured by the following EMAs. Participants had the option to reply to messages but were not explicitly asked to do so.

### 2.4 Measures

The primary EMA outcomes were desire to live, desire to die, and urges for suicide, which were assessed during every EMA. Participants responded to the questions: “How much do you want to die right now?” (desire to die), “How much do you want to live right now?” (desire to live), and “How strong are your urges for killing yourself right now?” (suicide urges) using Visual Analogue Scale (VAS) from 0 to 10. Higher scores on the VAS indicated higher desire or urges. Their convenience and ease of rapid administration make them highly compatible with EMA research designs, facilitating efficient real-time data collection in naturalistic settings (McCormack et al., 1988; Wewers and Lowe, 1990). Past studies have validated the use of similarly worded VAS to assess suicidal ideation, intent, and urges in adult and military samples (Brown et al., 2025; Bryan, 2019; Goldney, 1979; McCormack et al., 1988; Rogers, 2021).

During the EMA blocks, we also assessed four potential mechanisms of action for CC based on prominent psychological suicide theories (Joiner, 2007; Klonsky and May, 2015; O'Connor and Kirtley, 2018): burdensomeness, entrapment, mattering, and personal and social responsibility.

Perceived burdensomeness is consistently cited as a predictor of suicidal ideation, reflecting the harmful impact of feeling like a burden to others. It was assessed using a slightly modified version of an item from the Interpersonal Needs Questionnaire (Van Orden et al., 2012)(“I felt that I was a burden to other people, or that they would be better off without me”) that has been used in previous EMA research (Eisenlohr-Moul et al., 2022). Participants responded to this item on a 7-point Likert scale, ranging from 1 (“Not at all true for me”) to 7 (“Very true for me”), during every EMA.

Entrapment is a key construct in the IMV model of suicide (O'Connor and Kirtley, 2018) and represents the psychological state of feeling trapped with no escape, which has been associated with suicidal intent. It was assessed using a single item from the scale (“I feel trapped”), and participants responded to this item on a Likert scale from 0 (“Not at all like me”) to 4 (“Extremely like me”) during every EMA.

Mattering is how an individual perceives their significance and importance to others, which directly counters feelings of insignificance and isolation. During the EMA period, measurements were taken once a day using the 5-item General Mattering Scale (GMS) (DeForge and Barclay, 1997), which included items such as “How much do other people depend on you?” and “How important do you feel you are to other people?” Participants responded to these items on a Likert scale from 1 (“Not at all”) to 4 (“A lot”), with higher total scores indicating increased feelings of mattering to others.

Personal and Social Responsibility is an individual's feeling of accountability towards their own and others' well-being. It speaks to an individual's sense of belonging within broader social systems and reinforces protective roles within their network. This was measured once a day using the 8-item Responsibility Scale (Arslan and Wong, 2021). The Personal Responsibility Subscale contained 4 items, such as “I discipline myself to make the best use of my time doing meaningful things,” and “I am conscientious in whatever I do, big or small” and the Social Responsibility Subscale contained 4 items such as “I am morally accountable for how I treat others” and “I am responsible to do my part to make the world a better place.” Participants scored these items on a Likert scale from 1 (“Strongly disagree”) to 5 (“Strongly agree”), with higher total scores indicating a greater sense of personal and social responsibility (Arslan and Wong, 2021).

In an effort to reduce participant burden of EMA while ensuring valid and reliable data collection, the study included a planned missingness design. Specifically, for the mattering, personal responsibility, and social responsibility scales, it was planned that participants would see one or none of these measures at EMA ping based on a random number generator to ensure that no systematic variables were influencing missingness. Previous simulation research provides strong evidence that planned missingness designs yield unbiased estimates and generate data that conforms to a missing completely at random (MCAR) assumption (Xu and Logan, 2024). This approach allows for missing data methodology, including multiple imputation and maximum likelihood estimation (Silvia et al., 2014).

### 2.5 Qualitative interview questions

The qualitative interview was based on a guide developed for a previous study (Hebert et al., 2022), which was shown to be effective in exploring the impact of the CC intervention. A sub-set of questions to assess participants' experiences with EMA was added based on existing research, concepts of interest to the study team, and observations made during the course of the pilot study (See Table A.2).

### 2.6 Safety monitoring

Participant suicide risk was managed using two versions of the University of Washington Revised Risk Assessment Protocol (UWRRAP) (Linehan et al., 2012; Reynolds et al., 2006; Ward-Ciesielski and Wilks, 2020). Quantitative assessments (surveys and EMA) use the UWRRAP-Online version, which was programmed to provide resources where appropriate throughout a REDCap flow that is activated if participants endorsed elevated levels of suicidal ideation, urges, or a recent suicide attempt for which they did not get treatment. The risk assessment/response is multi-tiered depending on the severity of a participant's score, including but not limited to:

• On-screen reminders about the National Suicide and Crisis Lifeline, the Crisis Textline, and instructions on how to reach the Veteran-specific crisis line
• In-survey prompts for participants to report how they would respond to negative feelings or suicidal thoughts, and whether they have any fun or distracting activities planned for the day
• Prompts to identify safety contacts
• Crisis line information (provided by email, text, or phone, depending on preference), along with study contact info and hours of availability

Qualitative interviews use the standard UWRRAP, which is designed for interview-based assessment.

### 2.7 Quantitative data analytic procedures

All quantitative data analyses were conducted with *R*. Given the pilot nature of the project and small sample size, analyses largely focused on descriptive and feasibility metrics of utilizing ecological momentary assessment in the context of CC delivery, with additional interpretation based on inferential statistics (e.g., paired-samples *t*-tests, within-subjects correlations, intraclass correlations). Multiple imputation, using predictive mean matching from the *mice* package in R, was utilized for missing data on general mattering, personal responsibility, and social responsibility in line with planned missingness designs (Rhemtulla and Little, 2012).

First, to examine changes from baseline to follow-up on suicide outcomes and mechanisms, mean differences between the first EMA of the first block and final EMA of the last block were explored with paired samples *t*-tests, as well as box plots, to determine if participants improved.^1^ These timepoints were selected to parallel a traditional pre-post assessment of change across the study period. We did not average observations across the first and final EMA blocks because a Caring Contact could be delivered during an EMA block; averaging across the block could therefore combine observations occurring both before and after a Caring Contact. Second, to examine the dynamic nature of suicide outcome and hypothesized mechanisms, first intra-class correlations were calculated, which quantifies the amount of variance attributed to within vs. between individuals over the course of the study period (range 0–1; higher values = more between-person variability, pointing to individual difference factors). Additionally, summary plots were created, one for each outcome (desire to die, urges for suicide, desire to live) and mechanism, plotting the mean (by month) as well as the intra-individual standard deviation. This approach allows for examination of differences in mean changes compared to changes in variability over the assessment period.

Finally, to examine the preliminary impact of mechanisms on suicide outcomes, within-subject repeated measures correlations were examined, and the magnitude of the correlation (along with corresponding 95% CI and *p*-value) was reported. Additionally, given the hypothesized dynamic relationship between the mechanisms and outcomes, a summary plot displaying these relationships over time was generated from overall participant mean values. To explore the potential impact of CC delivery on mechanisms and outcomes, CC delivery indicators based on the co-occurrence of a Caring Contact and EMA burst in month 3 were also incorporated into the summary plots.

To assess feasibility, summary EMA completion rates (number, duration) were reported along with qualitative findings related to completion of these assessments.

### 2.8 Qualitative data analytic procedures

Audio recordings of participant interviews were transcribed by a HIPAA-compliant transcription service, GMR Transcription. The codebook utilized for analysis was developed by the study that also established the interview guide (Klonsky and May, 2015). This codebook included deductive codes based on the interview guide and inductive codes that were determined after reviewing the interview data. The Rap-GAP method (Hsu et al., 2024) was utilized to analyze interview transcripts. Two members of the research team experienced in qualitative coding (AEE, AH) applied this existing codebook to a subset of four transcripts to establish inter-rater reliability. The researchers discussed, defined, compared, and refined the codes. Once consensus with codebook definitions and the application of codes was reached, all remaining transcripts were independently coded by the same two researchers (Bryan et al., 2020). Coded data was then sorted and analyzed by first creating interview matrices for each code, then developing memos for each code to identify emergent themes. Researchers AEE and AH then individually reviewed each memo's coding to ensure an accurate analysis.

## 3 Results

16 psychiatry patients from the OPC site and 14 veterans from the VSO site were recruited (Total *N* = 30; See Table A.1 for participant demographics). Of the 30 participants, 14 (7 from each site) completed a qualitative interview.

### 3.1 Changes in suicide outcomes across the study period

Examining changes from baseline to follow-up reveal changes in suicide outcomes. Mean differences between the first EMA of the first EMA block and last EMA of the last EMA block are displayed in Table B and Fig. 2. Specifically, observed mean differences indicated a decrease in suicide urges (*t* (16) = −1.25, *p* = 0.23) and desire to die (*t* (16) = −1.87, *p* = 0.08), but these differences were not statistically significant. We did, however, observe a statistically significant increase in desire to live (*t* (16) = 2.47, *p* = 0.03).

**Fig. 2:**
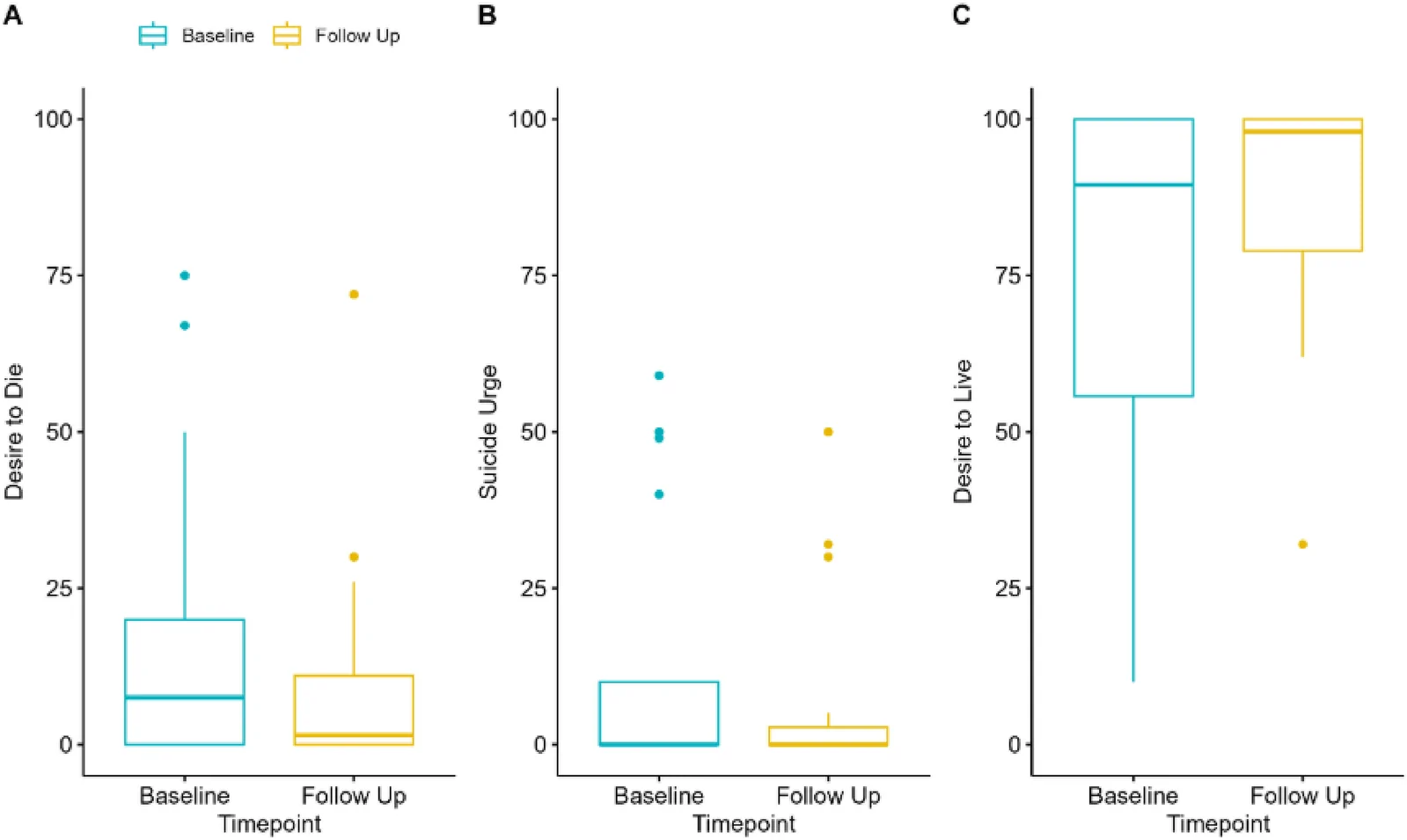
Baseline to Follow-Up Changes in Suicide Variables.

### 3.2 Variability in suicide outcomes and mechanisms

Intraclass correlation calculations (ICC) for all suicide outcomes and mechanisms were calculated and revealed differential patterns. For suicide outcomes, ICC values were as follows: desire to die – ICC = 0.75, suicide urges – ICC = 0.66, desire to live – ICC = 0.75. For suicide mechanisms, ICC values were as follows: entrapment – ICC = 0.88, perceived burdensomeness – ICC = 0.84, mattering – ICC = 0.06, personal responsibility – ICC = 0.35, social responsibility – ICC = 0.34. The results suggest high levels of between-person variability, indicating stability in responses over time in suicide outcomes, perceived burdensomeness, and entrapment, significant within-person variability in general mattering over time, and a moderate level of within- and between-person variability in personal and social responsibility over time.

Utilizing the time-varying EMA data, results indicated differential trajectories of outcomes and mechanisms dependent on examining mean scores or variability (intraindividual standard deviation; iSD) over time. Specifically, upon visual inspection, there is greater variability in the direction and magnitude of change for iSD than mean scores for suicide outcomes (see Fig. 3), which is supported by numerical change values (see Table D). Examination of the mechanisms, both visually and through quantitative data, suggests minimal differences in the direction and magnitude of change between mean scores and iSD over time (see Fig. 4A, Fig. 4B, Fig. 4C and Table E).

**Fig. 3:**
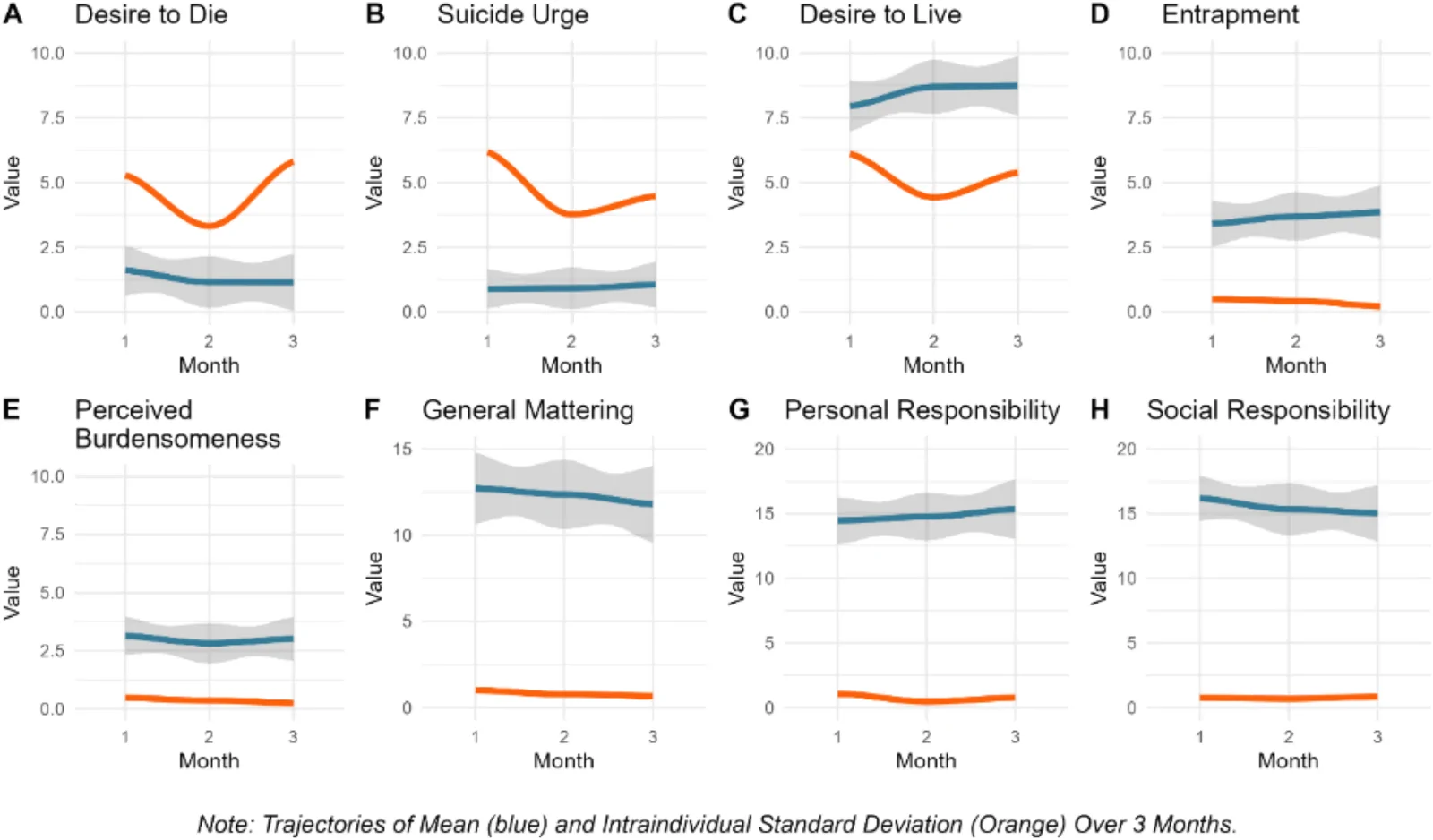
Mean (+SD) changes in (A) desire to die, (B), suicide urge, and (C) desire to live.

**Fig. 4A:**
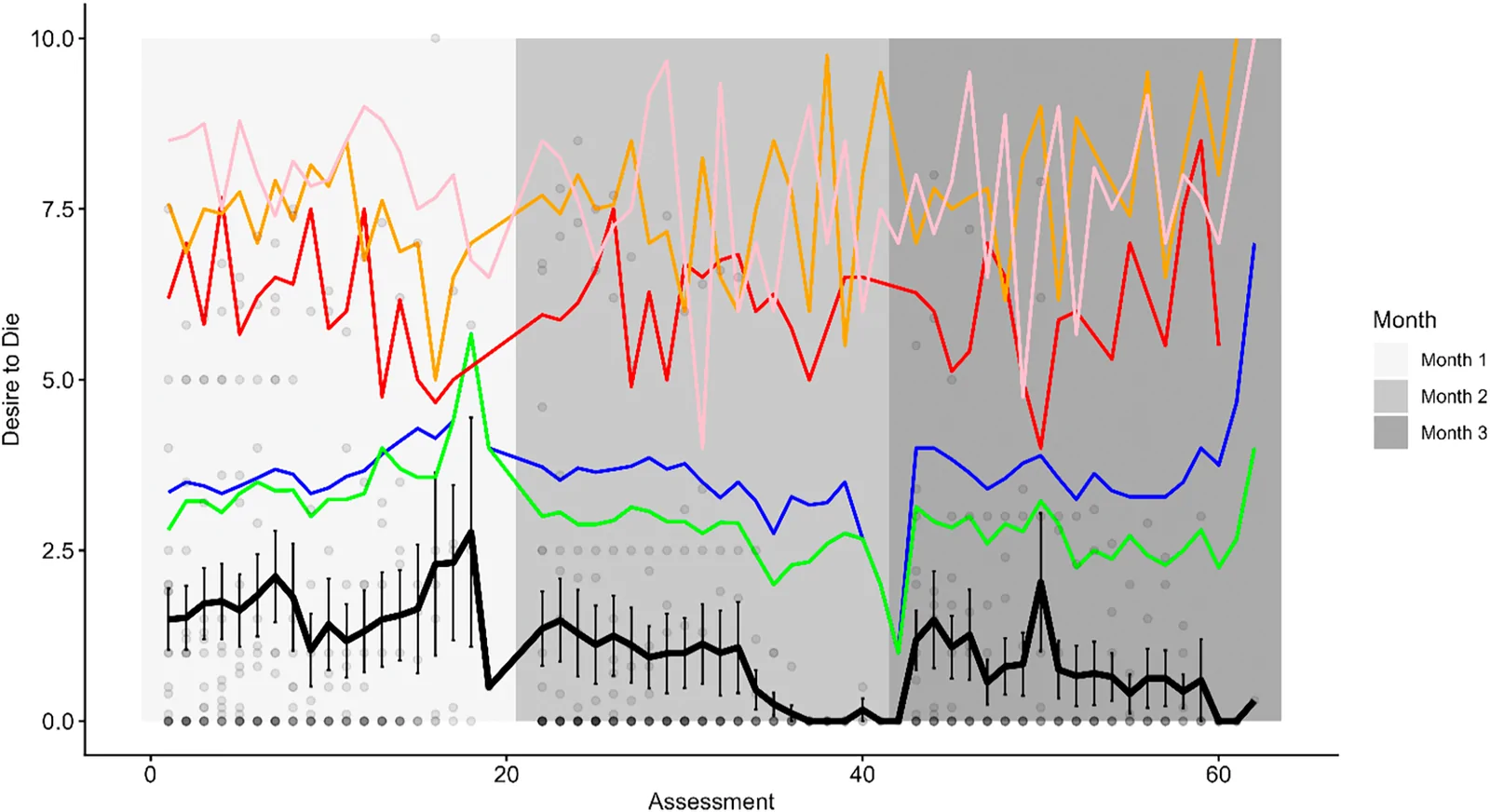
Summary Variability in Desire to Die by Month over time with Mechanisms **Note:** Mechanisms include entrapment (blue), perceived burdensomeness (green), mattering (red), personal responsibility (orange), and social responsibility (pink). Black line shows mean Desire to Die (+/− SD).

**Fig. 4B:**
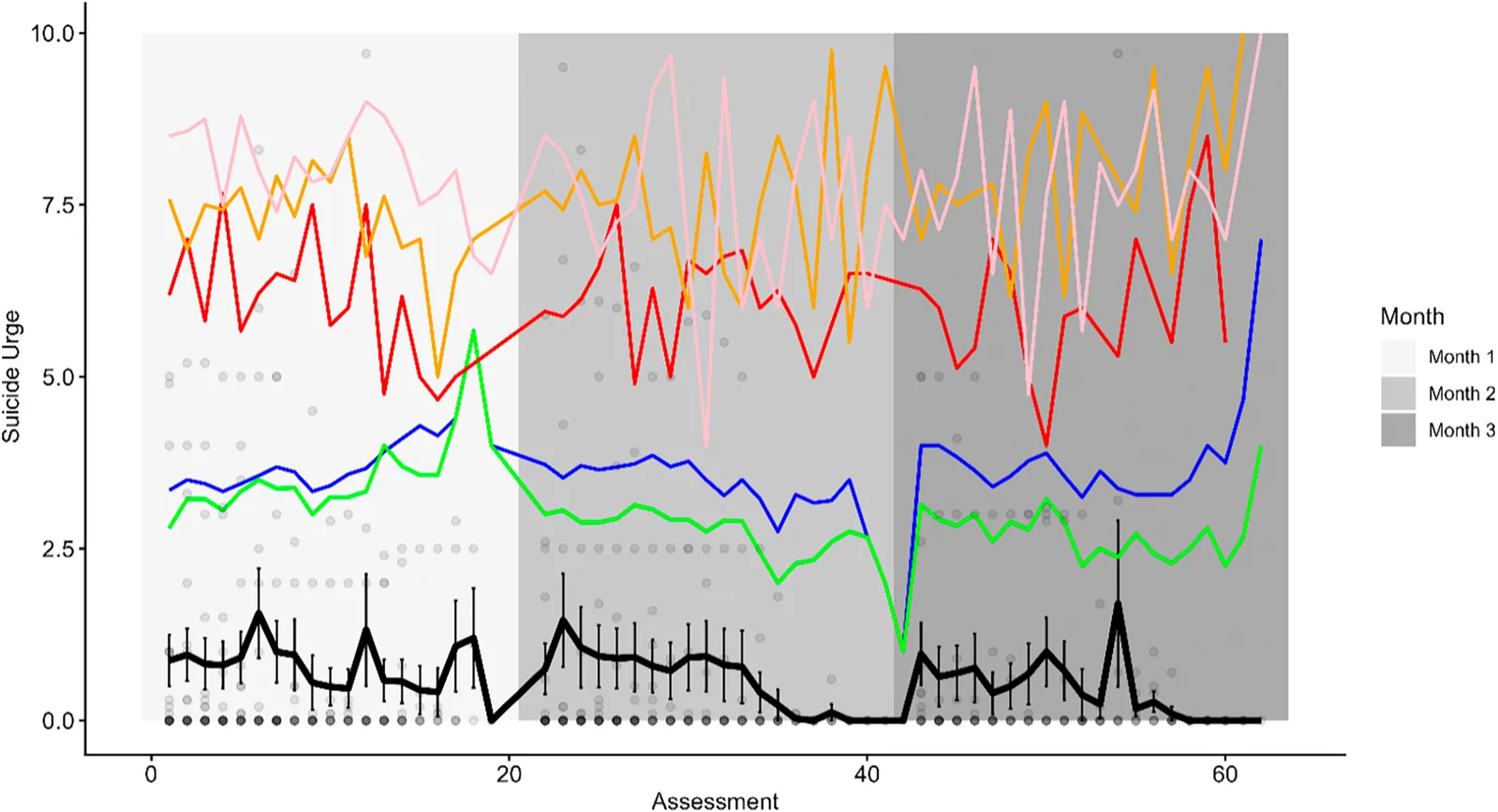
Summary Variability in Suicide Urge by Month over time with Mechanisms. **Note:** Mechanisms include entrapment (blue), perceived burdensomeness (green), mattering (red), personal responsibility (orange), and social responsibility (pink). Black line shows mean Suicide Urge (+/− SD).

**Fig. 4C:**
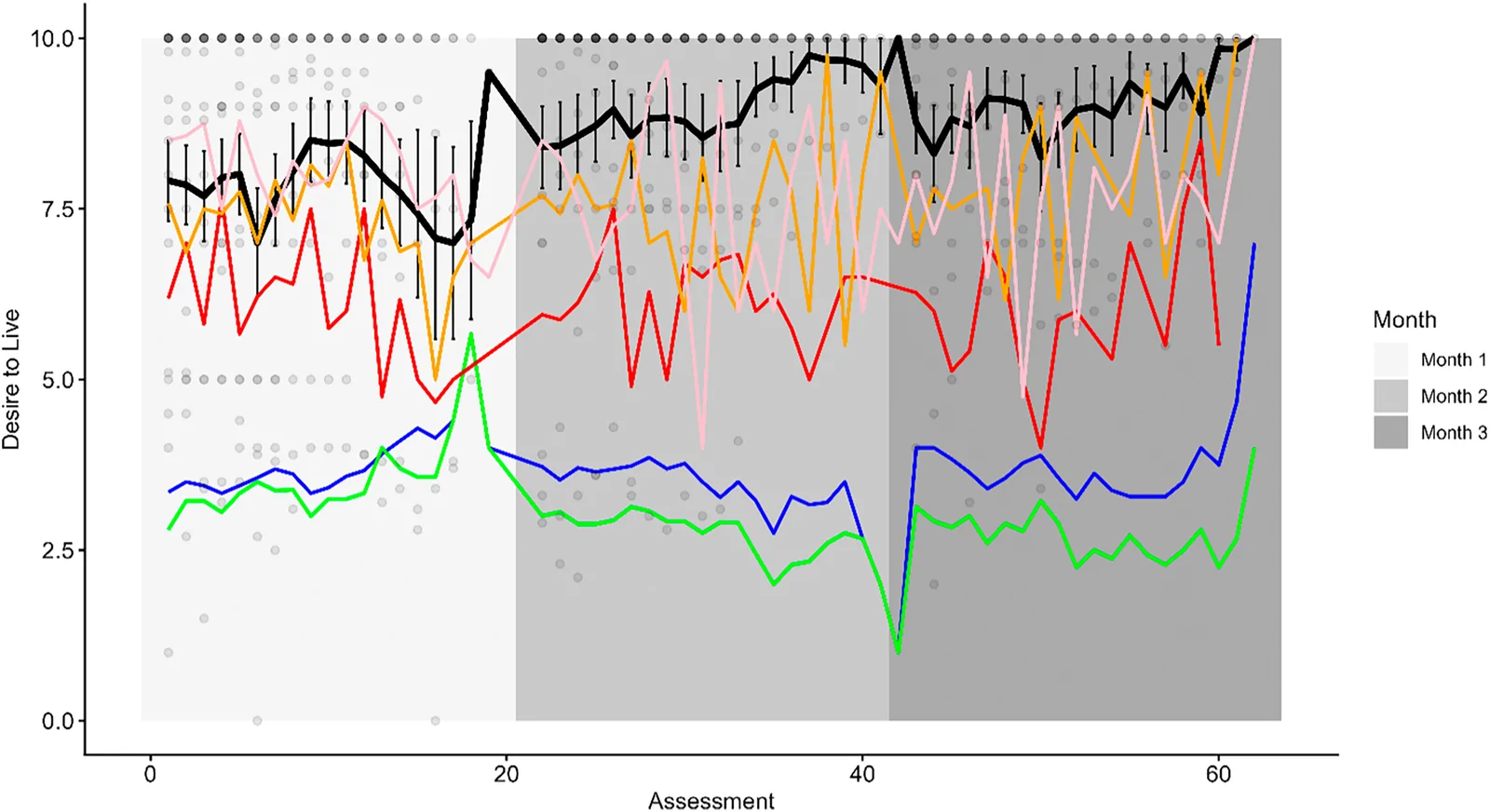
Summary Variability in Desire to Live by Month over time with Mechanisms. **Note:** Mechanisms include entrapment (blue), perceived burdensomeness (green), mattering (red), personal responsibility (orange), and social responsibility (pink). Black line shows mean Desire to Live (+/− SD).

Variability in suicide mechanisms, including perceived burdensomeness, entrapment, general mattering, social responsibility, and personal responsibility, can be seen in Fig. 4A, Fig. 4B, Fig. 4C. To assess the relationship between hypothesized mechanisms and outcomes, within-subjects repeated measures correlations highlight the associations between proposed mechanisms and suicide outcomes. Specifically, increased desire to die was significantly associated with perceived burdensomeness (*r* = 0.57, *95% CI* [0.52, 0.62], *p* < 0.001) and entrapment (*r* = 0.26, *95% CI* [0.18, 0.34], *p* < 0.001), but there were no significant associations with other mechanisms. Suicide urges were significantly associated with perceived burdensomeness (*r* = 0.34, *95% CI* [0.27, 0.42], *p* < 0.001) and entrapment (*r* = 0.27, *95% CI* [0.20, 0.35], *p* < 0.001), but there were no significant associations between general mattering, personal, or social responsibility. Finally, desire to live was negatively and significantly associated with perceived burdensomeness (*r* = −0.55, *95% CI* [−0.61, −0.49], *p* < 0.001) and entrapment (*r* = −0.24, *95% CI* [−0.31, −0.16], *p* < 0.001).

Finally, as a preliminary and illustrative example of the immediate and direct impact of a CC message on suicide outcomes and mechanisms, Fig. 5 displays suicide outcomes and hypothesized mechanisms before and after a Caring Contact message. Desire to live was generally higher following message delivery, with a brief decline followed by an increase and stabilization at higher values. Desire to die and suicide urges were generally lower following message delivery, aside from a brief increase before returning to lower values. Entrapment and perceived burdensomeness showed little apparent change surrounding message delivery and remained relatively low and stable.

**Fig. 5:**
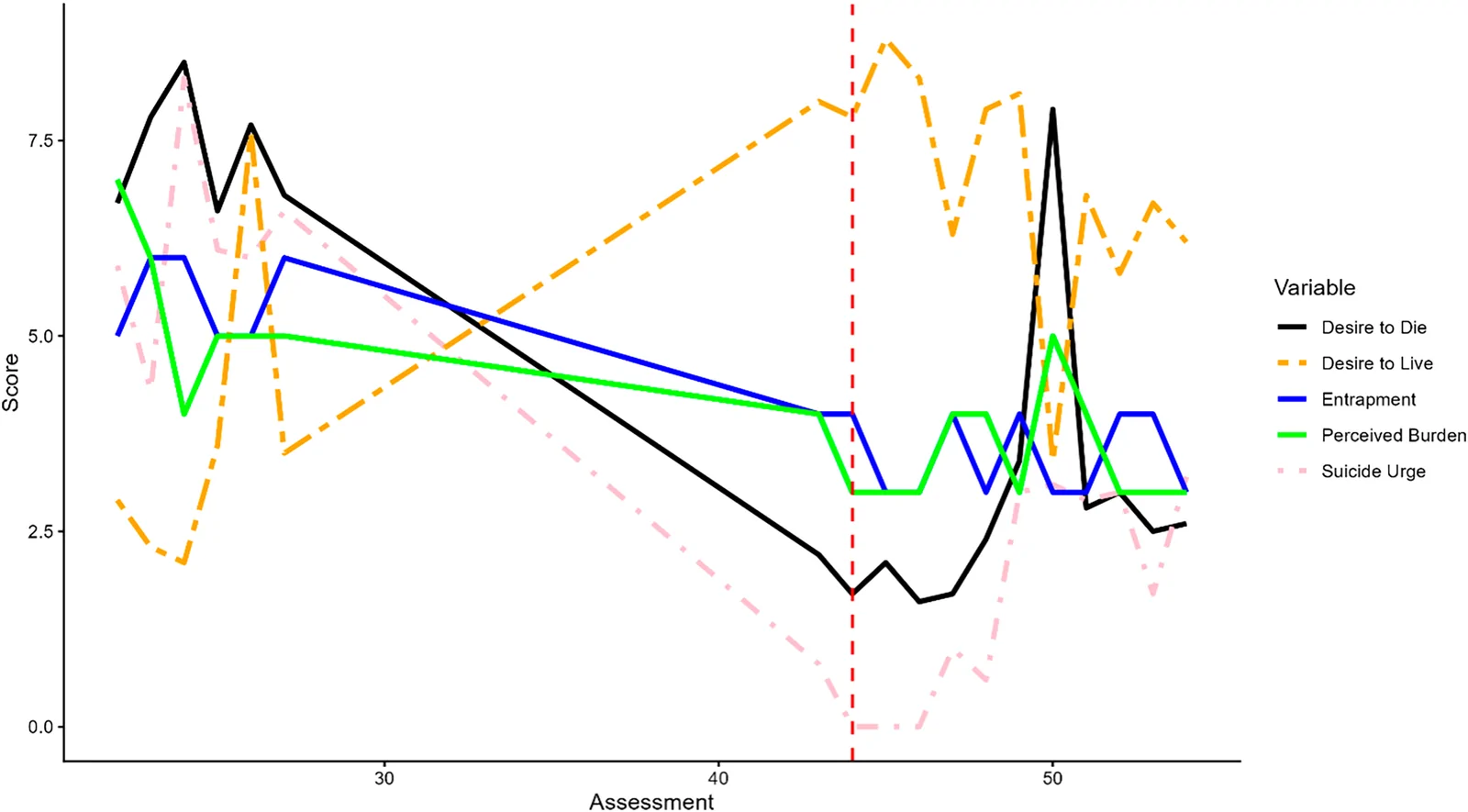
Longitudinal Trends in Suicide Outcomes and Mechanisms in Relation to Caring Contacts Message Delivery. **Note:** Data from example participant (n = 1) with a history of a suicide attempt. Red dashed vertical line indicates time of caring contacts message delivery.

### 3.3 EMA feasibility, usability, and acceptability

Feasibility metrics for EMA included compliance rates, or the average number of surveys completed for each month of EMA-burst data collection, as well as attrition for each month of data collection. Total average compliance rates for month 1 of data collection were 54.5%, 57.6% for month 2, and 51.1% for month 3. Of the 30 participants, 26 enrolled in EMA. Of these, 20 (66.7% of the full sample) completed at least one EMA assessment, including one participant who subsequently declined further assessments, while 3 (10.0%) enrolled but did not complete any EMA assessments. Six participants (20.0%) did not enroll in EMA. Separately, 2 participants (6.7%) declined further CC during the study. No participants formally withdrew from the study. Additional feasibility metrics, including the number of participants completing at least 1 EMA per month, as well as dropouts, are reported in Table C. The qualitative findings from participant interviews provided additional context for these feasibility metrics by identifying factors that facilitated EMA participation as well as barriers to completion. We identified four primary domains related to participants' experiences with the EMA protocol: Accessibility and ease of EMA participation, timing and prompt expiration as barriers to completion, practical and emotional burden of repeated assessments, and participant-identified opportunities for improving EMA. These domains, associated findings, and illustrative participant quotations are presented in Table F.

#### 3.3.1 Accessibility and ease of EMA participation

Of the participants who were interviewed (46.7%), a majority reported that the study surveys and EMAs were easy to understand and fill out, and they experienced no trouble navigating the technology required to complete them. Participants shared that the sign-up process was quick and easy, and none expressed any issues. Between signing up and the EMAs starting, participants reported that they expected to see the EMA questions, did not mind the delay, and remembered signing up for the surveys at the start of the study. One participant mentioned that they remembered the sign-up process being easy, but the instructions may have been overlooked due to their emotional state.

Most participants preferred receiving survey invitations by phone, specifically through text, and reported that they did not have any issues with accessing or completing the surveys. A significant majority of participants completed the surveys on their phones, and two participants reported completing the surveys on both their phones and their computers. Participants who completed the survey on their phones reported that it was more convenient. There were mixed feelings regarding the reception of invitations through phone and email, with some participants expressing that receiving invitations from both was excessive. In contrast, another participant shared that because their phone was not always functioning properly, receiving invitations through both methods was helpful.

#### 3.3.2 Timing and prompt expiration as barriers to completion

Most participants had a positive perception of the EMA and expressed that the EMAs were easy and relatively quick to complete. Some participants reported completing EMAs in 5 min or less, while others stated that it could sometimes take up to 15 min. About half of the participants reported that their primary issue with the surveys was that they expired, citing that these concerns were linked to poor timing and not being able to complete the surveys later after receiving the link or starting them due to busy schedules and work commitments. Some participants expressed being worried about not getting paid due to the time expiration and feelings of guilt for not completing all or most of the surveys.

#### 3.3.3 Practical and emotional burden of repeated assessments

A few participants shared that they had completed most or all of the surveys and commented that there were a lot of surveys sent to them but did not frame this in a negative or positive manner. Almost half of the participants had a negative perception of the EMAs, citing that they found them repetitive, while the longer baseline and follow-up surveys were excessively time-consuming, and there were too many surveys overall. Several participants shared that, while the surveys were logistically easy to complete, it was emotionally difficult to engage with the content and answer questions. Specifically, for those experiencing emotional distress or depressive episodes, the EMAs often intensified their negative emotions and reminded them of the lack of positive experiences in their lives.

Compared to the baseline and follow-up surveys, however, most participants preferred the shorter EMAs because they required less time and energy to complete; therefore, these were easy to complete while navigating life and other tasks outside of the study. A few participants had no preference between the EMAs and baseline/follow-up surveys, and one participant expressed enjoying the combination of both. Those who preferred the longer baseline and follow-up surveys or were neutral reported that this was due to their similarity to other surveys they've completed before, and in-depth content that allowed for deeper reflection than the EMAs.

#### 3.3.4 Participant-identified opportunities for improving EMA

Participants who had a negative perception of the EMA suggested improvements, such as shorter surveys, more time to access links, less frequent surveys but over a longer period of time, and incorporating resources, positive language, and reinforcement (ex., a message at the end of each survey). One participant suggested that there should be a wider range of answer choices in order to capture their current state more accurately.

Overall, participants had mixed feelings about the EMAs. The quantitative and qualitative findings provided complementary information regarding EMA feasibility, with qualitative findings identifying timing and expiration of prompts, competing demands, repeated assessment burden, and emotional difficulty as potential barriers to completion despite generally positive reports of EMA accessibility and usability.

## 4 Discussion

Overall, our observations and findings from this pilot study suggest that the EMA blocks have the ability to capture dynamic, intraindividual variability and temporal patterns that pre-post assessments may not be able detect, such as the timing, magnitude, and variability of changes within individuals. With the pre-post assessments, we found a significant increase in desire to live but we did not find significant decreases in suicide urges and desire to die. The EMA blocks, however, provided a more nuanced picture. We observed suicide urges to be relatively stable when examining mean levels across the three months, but found a modest within-person variability, highlighting their more dynamic nature. In contrast, desire to live and desire to die appeared to be relatively stable across time, with most of the differences seen between individuals. Specifically, desire to die seemed to decrease from Month 1 to Month 2 and then stabilized, while desire to live seemed to gradually increase from Month 1 to Month 3, both showing minimal intraindividual variability. Altogether, these findings suggest that desires to live and die may be relatively more stable than suicide urges. Furthermore, the observed decrease in the desire to die and its stabilization between Month 2 and Month 3, as well as the observed increase in desire to live were not mirrored by a similar decrease in suicide urges, which appeared to be relatively stable and even showed a slight increase in Month 3. This seems to suggest that desire to die, desire to live, and suicide urges are related but distinct processes and that suicide urges may persist even as desire to die decreases and desire to live increases. Existing studies applying dynamic systems theory further suggest that suicidal ideation often follows homeostatic patterns, exhibiting cyclical fluctuations that eventually return to a stable baseline (Bryan et al., 2025; Rogers et al., 2024; Rogers and Joiner, 2019). In addition, variability and instability in person-centered temporal patterns of suicidal ideation have been shown to prospectively predict suicide attempts (Bryan et al., 2019).

With regards to the hypothesized mechanisms for CC, the EMA blocks were able to capture changes over time with the magnitude of change appearing to be more pronounced for perceived burdensomeness, entrapment, and general mattering and less pronounced for personal and social responsibility. Entrapment and perceived burdensomeness seemed relatively stable across time, with most of the observed differences between individuals, whereas mattering, personal responsibility, and social responsibility seemed to fluctuate more dynamically within individuals. The intraindividual variability for most of these mechanisms appeared to decrease over time, suggesting there may be more day-to-day stability as the study progressed. Altogether, these observations suggest that entrapment and burdensomeness are relatively stable, mattering was highly dynamic, and personal and social responsibility fell in between.

Repeated-measure correlations were conducted to examine whether changes in hypothesized mechanisms were associated with changes in suicide urges, desire to die, and desire to live within individuals over time. As expected, changes in perceived burdensomeness and entrapment were both correlated with changes in suicide urges, desire to live, and desire to live. General mattering was uniquely correlated with changes in suicide urges, and personal responsibility was uniquely correlated with the desire to live. Fig. 5 visually illustrates that, following a Caring Contact delivery, participants' desire to live may continue to be elevated, though it appeared to dip briefly before rising again and stabilizing at higher values. Additionally, we noticed desire to die and suicide urges seem to decrease and remain low following a Caring Contact delivery and observed a brief spike before decreasing and leveling off at lower values. Entrapment and perceived burdensomeness did not appear to change in direct response to a Caring Contact delivery but seemed to remain low and relatively stable after. Due to the limited sample size and meaningful heterogeneity in implementation across sites during this pilot study, these observations should be viewed as illustrative rather than inferential, and replication is warranted in a larger, fully powered sample. We deliberately refrain from offering interpretation beyond descriptive patterns.

Regarding feasibility and acceptability, participants found the sign-up process quick and easy. While they appreciated receiving survey links via text, some felt that invitations through both text and email were excessive. As such, it may be helpful to tailor communication strategies to individual preferences to avoid overwhelming participants. Most participants reported that surveys were easy to navigate and preferred using mobile phones for convenience. They favored shorter, less frequent surveys, citing their practicality and lower time and effort burden. Although some appreciated the depth of longer surveys, most found shorter surveys better suited to their routines. Although monthly EMA compliance remained above 50%, qualitative findings provided important context for these rates. Participants generally described the EMA surveys as easy to navigate and technologically accessible, suggesting that usability itself was not a primary barrier to completion. Instead, common barriers that may have contributed to participants' inability to fully engage with the study included difficulty completing surveys before they expired, often due to poor timing or competing responsibilities. Furthermore, some participants noted that emotional barriers, such as distress during depressive episodes or negatively phrased survey content, sometimes hindered engagement.

Suggestions offered by participants for improving the implementation of future EMA and EMA blocks include eliminating expiration deadlines, reducing survey frequency, incorporating positive reinforcement, and offering more flexible and inclusive response options. Although eliminating expiration windows entirely would compromise the temporal specificity of momentary assessment, future studies could examine whether longer or more flexible response windows improve compliance while preserving the benefits of EMA. These findings reinforce the potential of EMA as a tool for real-time data collection but also emphasize the need for thoughtful adaptations to improve participant retention and engagement. Future studies should consider addressing the aforementioned logistical and emotional barriers, optimizing survey design, and integrating participant-centered features to improve feasibility and compliance.

This pilot study has several strengths that demonstrate the potential of EMA blocks as a valuable addition to methods used in suicide prevention research and clinical trials. First, the use of EMA blocks permitted the ability to capture more variability in suicidal ideation, urges, and related constructs than traditional pre-post assessments, providing a richer understanding of dynamic patterns in risk. While primarily descriptive, the method recorded whether changes occurred following the delivery of CC, demonstrating the feasibility of using EMA to track progress and mechanisms of change in future clinical trials. Incorporating EMA into clinical trials could offer a more detailed and nuanced understanding of the processes and mechanisms driving the interventions being tested. Mostly, the EMA process was fully automated, minimizing the labor demands on study staff and highlighting its scalability for larger studies and trials. The integration of qualitative data to assess the feasibility, acceptability, and scalability of EMA blocks enriched the study by providing valuable context to complement the descriptive quantitative findings. Participants shared insights about their overall experience, including the sign-up process, how many EMA prompts they recalled completing, how long the prompts took, issues encountered, missed prompts, and comparisons between EMA blocks and pre/post surveys. This qualitative feedback offered a deeper understanding of participants' experiences and engagement with this method, highlighting specific areas for refinement and improvement.

There are several limitations that must be considered when interpreting the findings. First, the small sample size and pilot nature of the study limit the generalizability of the results. While we primarily relied on descriptive data, we were able to conduct some paired-samples *t*-tests and repeated measures correlations, which provided insights into the relationships between desire to die, desire to live, suicide urges, and our hypothesized mechanisms. However, the small sample size and lack of robust inferential statistics means that these findings should be taken as exploratory and interpreted with caution. Because of the small sample size and lack of power to detect differences, non-significant findings may not directly indicate a lack of association, and replication is required. Second, our study recruited participants from two different clinical sites and followed their existing plan for delivering CC. The variation in how each site implemented CC (ie. the schedule and content of their messages, and response time to their clients), along with other site differences, may have influenced participants' experiences and outcomes. These site-specific factors were not fully accounted for in the analysis, given the small sample. However, allowing for variability between the two sites allowed us to examine different methods of implementation to inform future work. Third, comparisons of suicide outcomes across the study period relied on participants' first EMA assessment of the first block and final EMA assessment of the last block. These timepoints were selected to approximate pre-post measurement while avoiding averaging observations that could fall both before and after Caring Contact delivery within a block. However, because suicidal desire and urges are dynamic, individual EMA observations may reflect momentary variability and should not be interpreted as stable estimates of participants' beginning- and end-of-study levels. Finally, while we selected constructs such as perceived burdensomeness, entrapment, personal responsibility, social responsibility, and general mattering based on theoretical frameworks emphasizing the importance of connectedness in active suicidal ideation and desire, it is possible that other relevant mechanisms were not captured. Future research could explore additional mechanisms to ensure a more comprehensive understanding of the processes driving the impact of CC on suicide risk.

The repeated EMA block design that we piloted in this study fills a gap in the existing arsenal of methodological tools used in clinical trial research. It integrates EMA protocols focused on continuous monitoring over a brief time period and measurement burst designs. This integrated approach extends the utility of these two designs by allowing investigators to evaluate acute changes related to a treatment and their longer-term trajectories by capturing dynamic processes within days and larger shifts between months of an intervention. In addition, timing these EMA blocks to coincide with treatment phases and the delivery of a major treatment component may allow us to observe and test mechanistic hypotheses. With this pilot study, we demonstrated that this design is effective, feasible, and acceptable across 3 months, allowing for the detection of acute treatment effects and processes. This pilot study also provided insights that informed the design of a larger clinical trial where EMA blocks are delivered over the course of 12 months (Wright et al., 2025). This trial uses a significantly larger sample size, allowing for more robust analyses of variability and mechanisms of change over time. These future efforts aim to address many of the current study's limitations, offering deeper insights into the feasibility, acceptability, and scalability of EMA blocks.

## CRediT authorship contribution statement

**Keyne C. Law:** Conceptualization, Methodology, Investigation, Data curation, Project administration, Funding acquisition, Supervision, Resources, Software, Validation, Formal analysis, Writing – original draft, Writing – review & editing. **Andrew H. Rogers:** Conceptualization, Methodology, Project administration, Funding acquisition, Supervision, Resources, Data curation, Software, Validation, Formal analysis, Writing – original draft, Writing – review & editing, Visualization. **Annie Hoang:** Investigation, Data curation, Writing – original draft, Writing – review & editing. **Anna Evanson:** Conceptualization, Methodology, Investigation, Data curation, Formal analysis. **Barbara Wright:** Conceptualization, Methodology, Investigation, Data curation, Writing – review & editing. **Katherine Anne Comtois:** Conceptualization, Methodology, Investigation, Data curation, Project administration, Funding acquisition, Supervision, Resources, Software, Validation, Formal analysis, Writing – original draft.

## Funding details

This project was made possible through generous grant support from the Face the Fight Charitable Fund (grants 2022070003, 2023060005, 2024070014, and 2025060028).

## Declaration of competing interest

The authors report there are no competing interests to declare.

## Figures and Tables

**Table A.1:** Participant demographic characteristics.

	Recruitment Site	
Characteristic	VSO (Mean (SD) or %)	OPC (Mean (SD) or %)
Age		39.63 (10.53)
Gender Identity		
Man	57.1%	18.7%
Woman	42.9%	81.3%
Sexual Orientation		
Heterosexual or straight	78.6%	75.0%
Bisexual	7.1%	6.3%
Pansexual	7.1%	0.0%
Queer	0.0%	6.3%
Not listed	7.1%	0.0%
Prefer not to answer/Unknown	0.0%	6.3%
Race		
White	86.7%	75.0%
Black or African American	7.1%	6.3%
Korean		6.3%
More than one race	7.1% *	
	*n = 1 participant endorsed Black or African American and American Indian or Alaska Native–Cherokee	
I don't know		12.5%
Ethnicity		
Not Hispanic/Latino	50.0%	81.3%
Hispanic/Latino	50.0%	18.7%
Employment		
Working	42.9%	50.0%
Temporary laid off (sick leave, maternity leave)	7.1%	0.0%
Unemployed, looking for work		10.0% (3)
Disabled		10.0% (3)
Student		20.0% (6)
Other	14.3%	0.0%
Declined to answer		6.3%
Military Status		
Never served in the US Armed Forces		53.3% (16)
Current active duty		10.0% (3)
Past active duty		36.7% (11)
Current (past 2 week) Suicidal Ideation (PHQ-9)	28	
Yes		28.6% (8)
No		71.4% (20)
Suicide Attempt History	30	
Yes		23.2% (13)
No		76.8% (43)

**Table B:** Baseline to follow up changes in suicide outcomes.

	Baseline Assessment Mean (SD)	Follow Up Assessment Mean (SD)	% change
Suicide Urge	10.64 (19.28)	8.45 (17.05)	−20.61%
Desire to Live	77.82 (27.79)	88.25 (18.15)	+13.43%
Desire to Die	16.59 (22.4)	9.95 (17.8)	−40.02%

**Table C:** EMA Feasibility Metrics.

	Month 1	Month 2	Month 3
At least 1 per day completed (Perfect daily compliance)	20 ppl (66.7%)	18 ppl (60%)	15 ppl (50%)
Compliance…	54.5%	57.6%	51.1%
Dropouts	0 ppl	0 ppl	1 ppl

**Table D:** Mean and Intraindividual Variation (iSD) of EMA Suicide Outcomes.

	EMA Month 1	EMA Month 2	EMA Month 3
Desire to Die			
Mean	16.02	11.51	11.46
iSD	5.29	3.32	5.81
Suicide Urge			
Mean	8.92	9.08	10.50
iSD	6.18	3.77	4.48
Desire to Live			
Mean	79.53	86.98	87.36
iSD	6.11	4.43	5.39

**Table E:** Mean and Intraindividual Variation (iSD) of EMA Suicide Mechanisms.

	EMA Month 1	EMA Month 2	EMA Month 3
Perceived Burdensomeness			
Mean	3.14	2.82	3.01
iSD	0.48	0.35	0.25
Entrapment			
Mean	3.41	3.69	3.85
iSD	0.49	0.43	0.21
General Mattering			
Mean	12.72	12.36	11.78
iSD	1.01	0.77	0.67
Personal Responsibility			
Mean	14.45	14.75	15.35
iSD	1.06	0.45	0.77
Social Responsibility			
Mean	16.17	15.34	15.01
iSD	0.75	0.68	0.85

**Table F:** Qualitative interview domains and illustrative quotes.

Domain	Illustrative Quotes
Accessibility and ease of EMA participation	“*They were pretty easy to complete […] The questions were pretty straight[forward]. The scale is on a numerical 1 to 10 scale, so it wasn't very hard to fill out*.” “*[The] sign-up process was pretty easy. Basically, people calling me and registering once [my provider] communicated with your team that I was interested in participating. That was pretty easy.*” “*I wasn't surprised. I knew they were coming. [My provider] just said it would take a little bit for them to come […] I was just watching for them every day and then they finally showed up.*’ *“I felt like the signup was pretty easy… I was suffering pretty bad mentally. So, I probably wasn't reading everything like I should have.*” “*I rarely did them on the computer […I] mostly [used] my phone, just because I had it with me all the time.*” *“I opened my email, and I did the survey still on my phone though.”* *“I get why it was done, but [it was] a little bit overkill, having both the text messages and the emails fill up my inboxes […] for me it was more convenient to do the text messages.”* “*I would get a text and email… I had it set to go to both, just because I don't have the best phone, and it doesn't always notify me when I get an email, so I had it set to do both. And so that way, if one didn't come in, the other one would.*”
Timing and prompt expiration as barriers to completion	“*They weren't that long, and they were pretty easy to answer.*” “[*The EMA surveys] were a lot quicker. I want to say they took me, I feel like less than five minutes.*” “*Probably no more than 15* *min at the most*.” “*I think there was also the added pressure of having the surveys be monetized […] having the financial aspect tied to it makes the pressure of completing it a little bit higher, which is helpful to motivate those of us in the study to do the thing. But at the same time, having such a short time span in which I can complete the survey was a bit of a struggle, and it was almost always while I was commuting, while I was driving, in the middle of my workday, those sorts of things, which made completing the survey harder than I think was intended*.” “*The surveys have a time expiration on it, and I found that kind of frustrating, just because I'd be in the middle of something that I couldn't get to my phone to, for like a few hours. And then, when I went to click on the link, the survey was already expired. So, sometimes it's just kind of inconvenient timing, or that I wouldn't even be able to answer them*.”
Practical and emotional burden of repeated assessments	“*I do remember making it a priority whenever I did get the survey to try to answer them as promptly as possible*.” “*The frequency of the texts I felt was too frequent and the number of questions. The repetitiveness of the questions. I understand. I am fascinated by research, and I was glad to participate. I knew what the purpose was and why for the most part, but it did become a bit excessive.*” “*What I didn't like about it is that it was very repetitive. It felt like it was asking you the same questions every time and it got robotic after a while, as in my answers were all, ‘Yeah, pretty much the same. I feel the same way.’ And I get it. If my mood was changing, it would give me a reason to change it up, but I was[in a] pretty steady state.*” “*If I did [have issues with the surveys], it was probably because of my mood. If I was just really, really depressed, and I just didn't want to deal with it. But for the most part, I responded to almost every survey*.” “[…] *the content of the [EMA] surveys was a little challenging, because I was in a pretty active and severe depressive episode. Asking about, ‘What is one thing you have to look forward to?’ And it's like, ‘Literally, nothing. I'm so depressed, my job sucks, everything is terrible,’ was usually my response in some way. That was my internal response. When that was the case, I would just leave that question blank because I'm pretty sure it was optional*.” “*[…] the [EMA] surveys would have been easier for me to participate in because I do work, and I go to physical therapy and have other things.*” “*Participant: The [EMA surveys] were more of cursory surface questions, where the [baseline/follow-up surveys], I thought, were a little more in depth and nuanced in what they were getting at. I fully realize that a lot of these questionnaires are essentially the same question, just different vernacular, and I noticed on the [baseline/follow-up surveys], it was more nuanced, trying to gage the difference in answers to get a more well-rounded look.* *Interviewer: Did you like either one better than the other between those two types?* *Participant: Not particularly*.” “*[…] it was a good mix of both. I mean, depending on the timeframe, of course, the stressors throughout the day, depending on my mood. So, [the baseline/follow-up surveys] took me away, which was good because I needed a break from certain aspects [of my life] that were going on. And then once I got that taken care of, the [EMA surveys] came in at the perfect time.*” “*I like the [baseline/follow-up] surveys. They were pretty classic, really familiar set up to different types of surveys that I've done before as part of my general depression and anxiety treatment.*”
Participant-identified opportunities for improving EMA	“*[…] if there's a way to make them a little shorter.*” “*The only thing I would change is not to put a time limit on the surveys. Or if they have to, then put a longer one. Like put at least a 24-h time limit on the surveys*.” “*Maybe spread it out over longer periods and give people more time for the surveys. ‘Cause I would've been happy to do the survey at the end of the day once a day* versus *four times a day.*” “*I think it would've been nicer if some of the questions had a little bit more of a positive spin on them… just because of where I was at in my life. I was in a really disenfranchised position where people were not listening to what I had to say, and [the questions] did make me feel really bad*.” “*I know that you are asking people questions to judge where they are on the scale of depression and stuff like that […] if there's a way to put some sort of positive reinforcement after someone knows how bad they are on a scale… For me, personally, I would like a positive message, but for other people, I think it would be important to have resource links*.” “*[…] the answer [options] that they have, I felt like I was in between on some of those, if that makes sense. It would be either ‘yes, a lot’ or ‘somewhat’ or ‘a little’ or it was something like ‘always’ […] And I just wanted one that said sometimes or occasionally. And there wasn't that answer. It was like you had to go to one extreme or the other, or you had to be totally neutral […] I couldn't pick the accurate level of what I wanted to answer because it wasn't given.*”

## Footnotes

1 Given limitations in what assessments were included in the baseline and follow up assessments, perceived burdensomeness and entrapment were assessed using the full scales at baseline and follow up, and the suicide outcomes (desire to die, suicide urge, desire to live) were examined as differences between the first EMA assessment and the last EMA assessment of those constructs.
