# Supplementary material for: Piloting an ecological momentary assessment block design to examine suicide outcomes and mechanisms in a caring contacts trial
Source: Internet Interv. 2026 Sep 30;46:100995. doi: 10.1016/j.invent.2026.100995 (PMC13656861; doi:10.1016/j.invent.2026.100995)
Supplement: Supplementary file 1 [file mmc1.docx]

**Supplemental Table A.2.**
Qualitative Interview Questions

- Over the 3 months you participated in the study, you also received invitations to complete quick check-ins/brief surveys online. How did answering those questions go for you?
- How did you complete those surveys? (i.e., via phone or computer)
- Did you have any trouble completing the surveys?
- How was the sign-up process for you?
- How did it feel to get the survey invites a few weeks after you signed up?
- When did you expect to get them?
- Did you remember they were going to come/Were you surprised? Why/why not?
- About how many surveys do you remember being invited to complete?
- About how many questions do you remember being in each survey?
- About how long do you think it took to complete each survey?
- Did you ever miss a survey? How did you feel about that? How would increasing the time help?
- How do you remember getting the invitations? Did you like the invitations better from one source vs another? (i.e., via text vs. Email)
- How would you compare the longer surveys you did at the beginning and end of the study compared to the brief surveys?
- Anything else you’d like to add about your experience completing those brief surveys?
- Can you tell me how you are feeling about your life now?
